# Risk factors and nomogram for gallbladder stones in patients with type 2 diabetes mellitus: a retrospective study based on LASSO regression

**DOI:** 10.3389/fendo.2026.1908086

**Published:** 2026-09-24

**Authors:** Yang Zhao, Rui Su, Jun Li, Hong-Fang Tuo

**Affiliations:** 1 Central Hospital, Tianjin University, Tianjin Institute of Hepatobiliary Disease, Tianjin, China; 2 Department of Hepatobiliary Surgery, Hebei General Hospital, Shijiazhuang, China; 3 Gastroenterology Department, Tianjin Fourth Central Hospital, Tianjin, China

**Keywords:** gallbladder stones, LASSO regression, nomogram, prediction model, type 2 diabetes

## Abstract

**Objective:**

To investigate factors associated with gallstone occurrence in patients with type 2 diabetes and to establish a nomogram for predicting gallstone presence in this population, aiming to support early risk stratification and prevention-oriented management.

**Methods:**

This retrospective study enrolled consecutive patients who visited the Hepatobiliary Surgery and Endocrinology departments from September 2023 to December 2024. Clinical data were collected via electronic medical records. After eligibility assessment and data collection, the dataset was divided chronologically into a training cohort (September 2023 to August 2024) and an internal temporal validation cohort (September to December 2024), both from the same institution. The model was developed in the training cohort and evaluated in the training and internal temporal validation cohorts using the area under the receiver operating characteristic curve (AUC), Brier score, calibration curves, calibration intercept, calibration slope, and decision curve analysis (DCA).

**Results:**

After LASSO and multivariable logistic regression analyses, age, hypertension, fasting blood glucose, triglycerides, very low-density lipoprotein cholesterol (VLDL-C), and apolipoprotein A1 (ApoA1) were retained in the final model. The model showed moderate discrimination, with AUCs of 0.713 (95% bootstrap CI 0.669–0.758) in the training cohort and 0.707 (95% bootstrap CI 0.630–0.781) in the internal temporal validation cohort. The corresponding Brier scores were 0.166 and 0.180. Calibration intercepts were 0.000 and −0.063, and calibration slopes were 1.000 and 0.872, respectively. Calibration curves and decision curve analyses were also examined.

**Conclusion:**

Patient age, hypertension, fasting blood glucose, triglycerides, VLDL-C, and ApoA1 were identified as predictors of gallstone occurrence in patients with type 2 diabetes. The nomogram showed moderate discrimination and may support risk stratification in this population, but its clinical utility requires external validation.

## 1 Introduction

Diabetes mellitus is a metabolic disorder primarily characterized by chronic hyperglycemia. Its occurrence is mainly associated with insufficient insulin secretion, impaired insulin action, or a combination of both abnormalities (1). Epidemiological data indicate that approximately 537 million adults worldwide currently have diabetes, with type 2 diabetes (T2DM) being the most common type. It is projected that by 2045, the total number of patients will increase to 783 million. Additionally, approximately 352 million people have impaired glucose metabolism, such as impaired fasting glucose or impaired glucose tolerance, and among this group, about 5% to 10% progress to type 2 diabetes each year (2). T2DM, formerly known as “non-insulin-dependent diabetes” or “adult-onset diabetes,” accounts for approximately 90%–95% of all diabetes cases. Its main characteristics are relative insulin insufficiency and varying degrees of peripheral insulin resistance rather than absolute insulin deficiency (3). In addition to having a significant impact on glucose metabolism, T2DM is often accompanied by damage to multiple systems and is closely related to the occurrence of various complications, mainly involving the circulatory, nervous, and digestive systems (4).

Gallbladder stones (GS) represent a prevalent digestive system disease in clinical practice, with a prevalence rate of approximately 10%–20% among the adult population (5). Based on composition, approximately 80% of GS are cholesterol stones, which are mainly formed by the precipitation and deposition of supersaturated cholesterol. Among other types, pigment gallstones account for about 10%–15% and are primarily composed of calcium bilirubinate, and are often associated with the deposition of unconjugated bilirubin, commonly seen in chronic hemolytic diseases. Brown pigment stones account for about 5% and are usually closely related to biliary tract infections (6). Clinically, approximately 70% of patients with GS are asymptomatic at diagnosis, and a significant proportion can remain asymptomatic for a long time (7). However, as the disease progresses, some patients may gradually experience symptoms such as nausea, upper abdominal cramps, diarrhea, and loss of appetite. Without timely intervention, GS can further lead to a series of serious complications, including cholecystitis, common bile duct stones, and pancreatitis (8). In severe cases, the condition may become life-threatening.

Although the conventional perspective suggests that GS occurrence is mainly related to factors such as obesity, high-fat diet, pregnancy, and high estrogen levels, emerging evidence consistently indicates that compared to the general population, the risk of GS is significantly increased in patients with T2DM (9, 10). From a pathophysiological perspective, this association may be mechanistically linked to insulin resistance. Insulin resistance not only causes lipid metabolism disorders but also further destabilizes bile components. Cross-sectional evidence has also linked hyperinsulinemia with gallstone characteristics, although the direction and causality of this association remain uncertain (11).

In addition, patients with T2DM often experience diabetic autonomic neuropathy, which can lead to impaired gallbladder contraction and delayed emptying, resulting in bile stasis and further increasing the risk of stone formation. Although GS is relatively common in the T2DM population, underdiagnosis or inadequate recognition still occur in clinical practice. Currently, the diagnosis of GS mainly relies on imaging examinations, among which abdominal ultrasound is the most widely used. This method has high diagnostic accuracy, with a sensitivity of approximately 95% and a specificity close to 100%, and is therefore considered the preferred method for diagnosing GS (12).

At present, the risk factors associated with GS occurrence in T2DM patients remain incompletely elucidated. In recent years, researchers globally have conducted numerous studies attempting to identify potential influencing factors for GS in this population (13, 14). However, previous studies have mostly relied on conventional statistical approaches, and their results still have certain limitations in terms of interpretability. For instance, using only traditional logistic regression models for risk factor screening may be confounded by multicollinearity among variables, especially when many binary variables are present. This issue is particularly pronounced in such cases, thereby preventing some potentially important factors from being effectively identified.

LASSO penalized regression is a predictive modeling method well-suited to the data structure of this study. It introduces a penalty function into the regression model to shrink variable coefficients, thereby mitigating overfitting to a certain extent (15, 16). LASSO is a regression technique that combines variable selection and regularization, which can improve the predictive performance of the model while enhancing its interpretability. In this method, the regression coefficients are shrunk toward zero under the influence of the penalty term, and some coefficients can even be compressed to zero, thereby achieving variable selection and dimensionality reduction. Moreover, LASSO regression offers distinct advantages in dealing with multicollinearity, especially when independent variables are highly correlated. By introducing a penalty term based on the absolute value of the coefficients, the model can automatically remove redundant predictors, making the resulting model more parsimonious and robust (16).

This study used retrospective clinical data and employed LASSO regression combined with multivariate logistic regression to systematically analyze predictors of GS occurrence in patients with T2DM and to construct a risk prediction model. Compared with conventional univariate statistical methods, this study substantially mitigated the impact of multicollinearity on model stability through LASSO-based variable screening and enhanced the robustness of variable selection. The constructed nomogram model not only permits quantitative assessment of candidate predictors but may offer an intuitive adjunctive risk-assessment tool after external validation for clinical practice. The model was validated using multiple complementary metrics including ROC curves, calibration curves, and DCA. The results showed that the model demonstrated moderate discriminative performance and potential decision-analytic utility, but routine clinical benefit remains unproven.

## 2 Materials and methods

### 2.1 Research subjects

This study is a retrospective cohort study. The study protocol was approved by the hospital’s ethics committee (Approval No. (2026) Scientific Ethics Review No. (029)). By screening data from the electronic medical record system of Hebei General Hospital and combining expert opinions with relevant literature, 22 candidate indicators for constructing a prediction model were initially identified. Clinical data were collected from hospitalized adults with T2DM who were treated in the Departments of Endocrinology and Hepatobiliary Surgery at Hebei Provincial People’s Hospital from September 2023 to December 2024. Gallstone status was assessed by abdominal ultrasonography. A total of 883 patient records were screened for eligibility. No records were excluded after eligibility assessment because of malignancy, severe liver or kidney dysfunction, autoimmune disease, or incomplete clinical data; therefore, 883 patients were included in the final analysis. The flowchart is shown in **Figure 1**. The included patients were divided chronologically into a training cohort (September 2023 to August 2024) and an internal temporal validation cohort (September 2024 to December 2024), both from the same institution. The prediction model was developed using the training cohort and applied to the later cohort. Candidate variables were restricted to demographic, clinical, biochemical, and comorbidity measures that were routinely recorded in the electronic medical record. HbA1c, diabetes duration, antidiabetic medications (particularly GLP-1 receptor agonists and insulin), lipid-lowering therapy, rapid weight loss, dietary habits, physical activity, family history, female reproductive factors, and previous bariatric surgery were not consistently available as standardized variables and were therefore not included. Inclusion criteria were as follows: (1) age ≥ 18 years; (2) definite diagnosis of T2DM; (3) abdominal ultrasound assessment available for GS status; (4) complete candidate-predictor data; and (5) adequate mental status and normal communication ability. Exclusion criteria comprised (1) malignant tumors; (2) severe hepatic or renal dysfunction; (3) autoimmune diseases.

**Figure 1:**
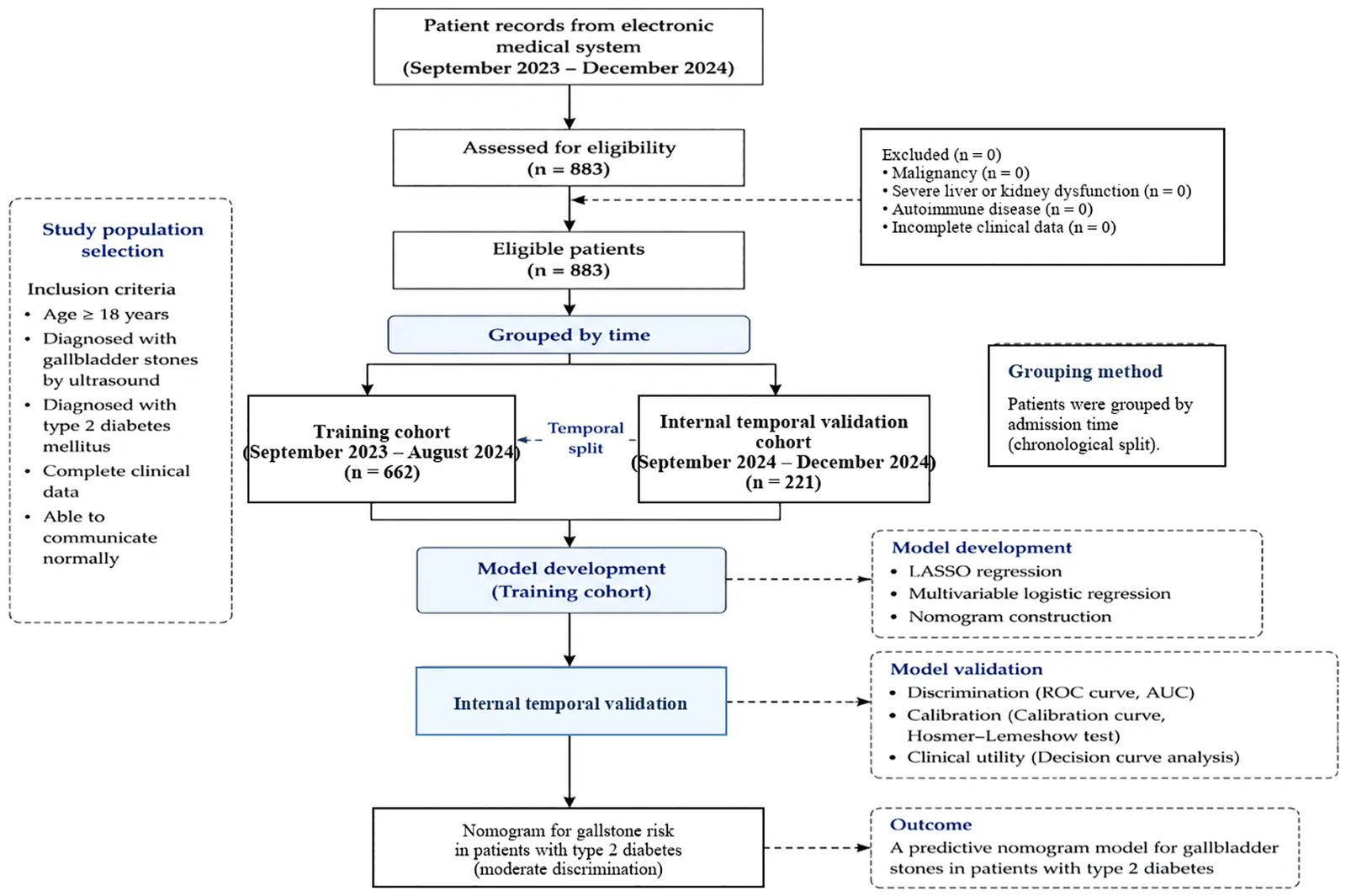
STROBE-style flowchart of patient selection and chronological cohort allocation.

### 2.2 Anthropometric and imaging measurements

Body weight and height were measured using a scale (HNH-219, Shenzhen Shuangjia Medical Technology Co., Ltd.), with patients wearing light clothing and no socks. Body mass index (BMI) was calculated as weight in kilograms divided by the square of height in meters. GS status was assessed by experienced clinicians using a color Doppler ultrasound scanner (EPIQ7C, Philips Ultrasound, USA) and standard abdominal ultrasonography. All subjects were required to fast before the examination. The examination was performed in the supine position, and the liver, gallbladder, pancreas, and spleen were examined in sequence. The presence of a stable, hyperechoic mass within the gallbladder lumen was diagnostic of GS (17). T2DM was defined according to the standards set by the American Diabetes Association (3). Specifically, the diagnosis of T2DM was based on (1) self-reported physician-diagnosed T2DM, (2) FBG ≥ 7.0 mmol/L (126 mg/dL), (3) use of antidiabetic drugs, and (4) hemoglobin A1c (HbA1c) ≥ 6.5% (48 mmol/mol).

### 2.3 Biochemical measurements

Biochemical indicators included total bilirubin (TBIL), alanine aminotransferase (ALT), aspartate aminotransferase (AST), gamma-glutamyl transferase (GGT), FBG, uric acid (UA), total cholesterol (TC), triglycerides (TG), high-density lipoprotein cholesterol (HDL-C), low-density lipoprotein cholesterol (LDL-C), very low-density lipoprotein cholesterol (VLDL-C), and apolipoprotein A1 (ApoA1). All serum biochemical parameters were measured using an automated biochemical analyzer (TBA-FX8, Canon Medical Systems (Dalian) Co., Ltd.).

### 2.4 Variable selection using LASSO regression

Variable selection was performed using R software (version 4.4.3). LASSO regression analysis was performed using the *glmnet* package in R to select the optimal subset of predictor variables. This technique applies penalty constraints to the model parameters in the training set, causing the regression coefficients of some variables to shrink to zero, thereby reducing prediction error. After shrinkage, variables with non-zero penalized coefficients were retained as candidate predictors because they contributed predictive information to the penalized model. In the LASSO regression analysis, a fixed random seed was used and 10-fold cross-validation was performed to identify the penalty parameter for variable selection. Lambda.min was selected because it yielded the minimum cross-validated deviance and prioritized predictive information during model development. This choice was appropriate because the primary aim of this study was to develop a model with predictive utility rather than to minimize the number of variables at the initial screening stage. The 22 candidate variables included correlated metabolic and clinical measures; therefore, using lambda.min reduced the risk that the stronger penalty associated with lambda.1se would prematurely remove weak-effect or correlated predictors that might contribute complementary predictive information. Lambda.1se would generally yield a more parsimonious model, but may exclude weaker or correlated predictors and was not selected as the primary criterion. Variables with non-zero coefficients at lambda.min were retained for a second-stage multivariable Logistic regression, which provided additional statistical screening and produced the final six-predictor model. We analyzed 22 features, including smoking (Smoke, binary, yes (1), no (0)), alcohol consumption (Alcohol, binary, yes (1), no (0)), age (Age, continuous), gender (Gender, categorical), BMI (continuous), hypertension (Hypertension, HTN, binary, yes (1), no (0)), fatty liver disease (Fatty liver disease, FLD, binary, yes (1), no (0)), gallbladder polyp (Polypoid lesion of gallbladder, PLG, binary, yes (1), no (0)), coronary heart disease (Coronary heart disease, CHD, binary, yes (1), no (0)), disease course (Course of disease, CD, continuous), TBIL (continuous), ALT (continuous), AST (continuous), GGT (continuous), FBG (continuous), UA (continuous), TC (continuous), TG (continuous), HDL-C (continuous), LDL-C (continuous), VLDL-C (continuous), and ApoA1 (continuous).

### 2.5 Multivariate logistic regression model development

The relevant risk variables selected by LASSO regression were incorporated into multivariable logistic regression, and odds ratios (ORs), 95% confidence intervals (CIs), and P values are presented. Independent predictors with P < 0.01 were selected for the final model. The recovered training cohort contained 161 GS events. The approximate events-per-variable (EPV) was 161/21 = 7.7 at the LASSO-retained candidate stage and 161/6 = 26.8 for the final six-predictor model. Model-performance uncertainty was quantified using 2,000 nonparametric bootstrap resamples, and the training-derived model was applied unchanged to the later same-institution cohort. Based on the multivariable results, a nomogram for GS occurrence in patients with T2DM was constructed.

Reporting guideline compliance. The manuscript was revised with reference to the Transparent Reporting of a multivariable prediction model for Individual Prognosis Or Diagnosis (TRIPOD) Statement (49). We report the study design and setting, participant selection and exclusions, outcome definition, candidate predictors, missing-data handling, model-development procedure, internal temporal validation, discrimination, calibration, decision-curve analysis, model limitations, and intended use. A completed TRIPOD item-by-item checklist is provided as **Supplementary Table S1**; items that are not applicable to this retrospective single-center study or that require future external validation are identified explicitly.

### 2.6 Prediction model performance evaluation

We used complementary measures to evaluate model performance. Discrimination was assessed using the area under the receiver operating characteristic curve (AUC). The six-predictor logistic model was fitted in the training cohort, and its coefficients were applied unchanged to the internal temporal validation cohort. Overall prediction error was quantified with the Brier score. Calibration was evaluated using observed-versus-predicted risk plots, the Hosmer–Lemeshow test, calibration intercept, and calibration slope; ideal calibration intercept and slope values are 0 and 1, respectively. The calibration parameters were estimated from a logistic calibration model using the logit of predicted probability as the sole predictor. Two thousand nonparametric bootstrap resamples were used to estimate 95% confidence intervals for the AUC, Brier score, calibration intercept, and calibration slope. Decision curve analysis was used to estimate potential net benefit across threshold probabilities.

### 2.7 Statistical analysis

Missing-data handling was completed during data collection and eligibility screening. Records with any missing candidate variable were excluded before analysis; consequently, all 883 included patients had complete data for the analyzed variables. Continuous variables are presented as median (interquartile range), and categorical variables as frequencies and percentages. Between-cohort comparisons used Yates-corrected chi-square tests for binary variables and two-sided Mann–Whitney U tests for continuous variables. Spearman’s correlation coefficient was used to assess pairwise associations among candidate predictors. P < 0.05 indicated statistical significance for descriptive comparisons, while P < 0.01 was used for final multivariable predictor selection.

### 2.8 Patient selection flowchart

## 3 Results

### 3.1 Baseline characteristics of the study cohort

A total of 883 patients were included: 662 in the recovered training cohort and 221 in the recovered internal temporal validation cohort. GS was present in 220 patients (24.92%), including 161/662 (24.32%) in the training cohort and 59/221 (26.70%) in the validation cohort (P = 0.537). **Table 1** presents the remaining demographic, clinical, and biochemical characteristics. GGT differed between cohorts (P = 0.010); the other reported comparisons did not reach P < 0.05.

**Table 1:** Baseline data table.

Variables	Total population n = 883 (%)	Training set n = 662 (%)	Validation set n = 221 (%)	*P*
GS				0.537
1	220 (24.92)	161 (24.32)	59 (26.70)	
0	663 (75.08)	501 (75.68)	162 (73.30)	
Smoke				0.969
1	173 (19.59)	129 (19.49)	44 (19.91)	
0	710 (80.41)	533 (80.51)	177 (80.09)	
Alcohol				0.540
1	129 (14.61)	100 (15.11)	29 (13.12)	
0	754 (85.39)	562 (84.89)	192 (86.88)	
Gender				0.800
1	531 (60.14)	396 (59.82)	135 (61.09)	
0	352 (39.86)	266 (40.18)	86 (38.91)	
FLD				0.321
1	424 (48.02)	311 (46.98)	113 (51.13)	
0	459 (51.98)	351 (53.02)	108 (48.87)	
PLG				0.076
1	49 (5.55)	31 (4.68)	18 (8.14)	
0	834 (94.45)	631 (95.32)	203 (91.86)	
CHD				0.846
1	158 (17.89)	117 (17.67)	41 (18.55)	
0	725 (82.11)	545 (82.33)	180 (81.45)	
HTN				0.985
1	449 (50.85)	336 (50.76)	113 (51.13)	
0	434 (49.15)	326 (49.24)	108 (48.87)	
Age	60 (50, 68)	60 (50, 67)	60 (51, 70)	0.477
BMI	25.78 (23.46, 28.32)	25.79 (23.40, 28.20)	25.71 (23.65, 28.59)	0.447
CD	10 (4, 17)	10 (4, 17)	10 (2, 16)	0.263
TBIL	13.10 (10.10, 17.00)	12.90 (10.10, 16.70)	13.70 (10.20, 17.90)	0.256
ALT	18.50 (13.30, 27.65)	18.40 (13.12, 26.50)	18.90 (13.80, 29.70)	0.286
AST	18.80 (15.90, 24.60)	18.80 (15.80, 24.27)	19.40 (16.00, 25.80)	0.428
γ-GT	23.40 (16.25, 35.00)	22.75 (15.90, 33.88)	25.10 (17.60, 42.00)	0.010
FBG	8.22 (6.47, 11.14)	8.21 (6.45, 10.95)	8.25 (6.69, 11.98)	0.545
UA	306.00 (251.85, 371.90)	303.95 (248.42, 372.00)	309.30 (262.00, 371.60)	0.267
TC	4.70 (3.87, 5.59)	4.73 (3.87, 5.63)	4.58 (3.84, 5.47)	0.354
TG	1.35 (0.93, 2.23)	1.35 (0.92, 2.19)	1.37 (1.01, 2.44)	0.274
HDL-C	1.14 (0.98, 1.34)	1.15 (0.99, 1.35)	1.13 (0.98, 1.32)	0.262
LDL-C	2.85 (2.23, 3.45)	2.85 (2.24, 3.50)	2.86 (2.17, 3.32)	0.250
VLDL-C	0.65 (0.46, 0.87)	0.65 (0.47, 0.87)	0.64 (0.43, 0.89)	0.990
ApoA1	1.23 (1.06, 1.44)	1.23 (1.06, 1.44)	1.24 (1.06, 1.43)	0.808

### 3.2 Multicollinearity assessment

Pairwise associations among candidate predictors were assessed using Spearman’s correlation coefficients, as presented in **Figure 2**. Among the 22 variables, strong correlations (|r| ≥ 0.7) were observed for ALT with AST (r = 0.75, P < 0.001), TC with LDL-C (r = 0.93, P < 0.001), and HDL-C with ApoA1 (r = 0.82, P < 0.001). Moderate correlations (0.3 ≤ |r| < 0.7) included Smoke with Alcohol (r = 0.57, P < 0.001), BMI with TG (r = 0.33, P < 0.001), Age with CD (r = 0.44, P < 0.001), GGT with TG (r = 0.46, P < 0.001), Gender with UA (r = 0.30, P < 0.001), and Smoke with Gender (r = 0.36, P < 0.001). Additional weak-to-moderate correlations included FLD with ALT, TG, and GGT (r ≈ 0.27–0.33) and Gender with HDL-C and ApoA1 (r ≈ −0.25, P < 0.001).

**Figure 2:**
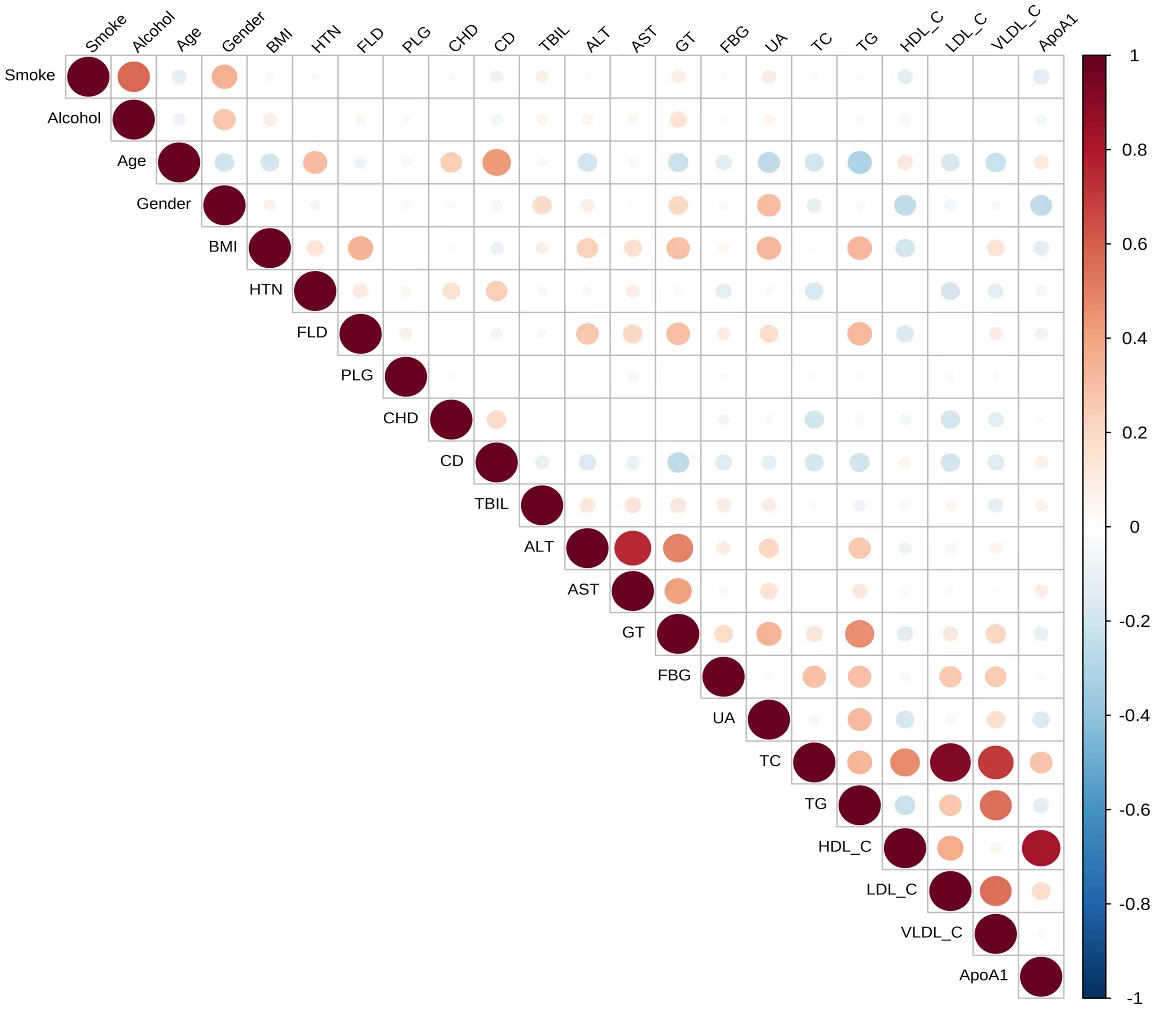
Correlation heatmap. This heatmap visually represents the correlation matrix of 22 variables. The color range in the heatmap goes from deep red indicating a positive correlation to deep blue indicating a negative correlation.

### 3.3 Prediction model development

#### 3.3.1 Variable selection via LASSO regression

LASSO regression was employed for variable selection to identify key predictors for inclusion in the model, with ten-fold cross-validation applied to enhance result robustness. **Figure 3** illustrates the optimal parameter selection and variable screening process. In **Figure 3A**, the x-axis shows log(lambda), the y-axis shows model deviance, and the numbers above each lambda value indicate the number of non-zero coefficients. The two vertical dashed lines correspond to lambda.min (the lambda value yielding minimum error) and lambda.1se (the lambda value one standard error from the minimum). Lambda.min was selected to minimize cross-validated deviance, corresponding to lambda = 0.001775742. In this prediction-oriented analysis, lambda.min was used to preserve potentially informative weak-effect or correlated predictors rather than to maximize initial sparsity. At this penalty, 21 of 22 candidate variables had non-zero coefficients; UA was shrunk to zero. These 21 variables were then entered into a second-stage multivariable logistic regression, which yielded the final six predictors. The retained variables were: Smoke, Alcohol, Gender, FLD, PLG, CHD, HTN, Age, BMI, CD, TBIL, ALT, AST, GGT, FBG, TC, TG, HDL-C, LDL-C, VLDL-C, and ApoA1. These 21 variables were then entered into multivariable logistic regression, which yielded the six predictors used in the final nomogram. In **Figure 3B**, as lambda increases, the penalty intensifies, leading to greater coefficient shrinkage and a progressive reduction in the number of selected variables. Each curve represents the trajectory of a variable’s coefficient, with different lambda values yielding different sets of predictors and their coefficients.

**Figure 3:**
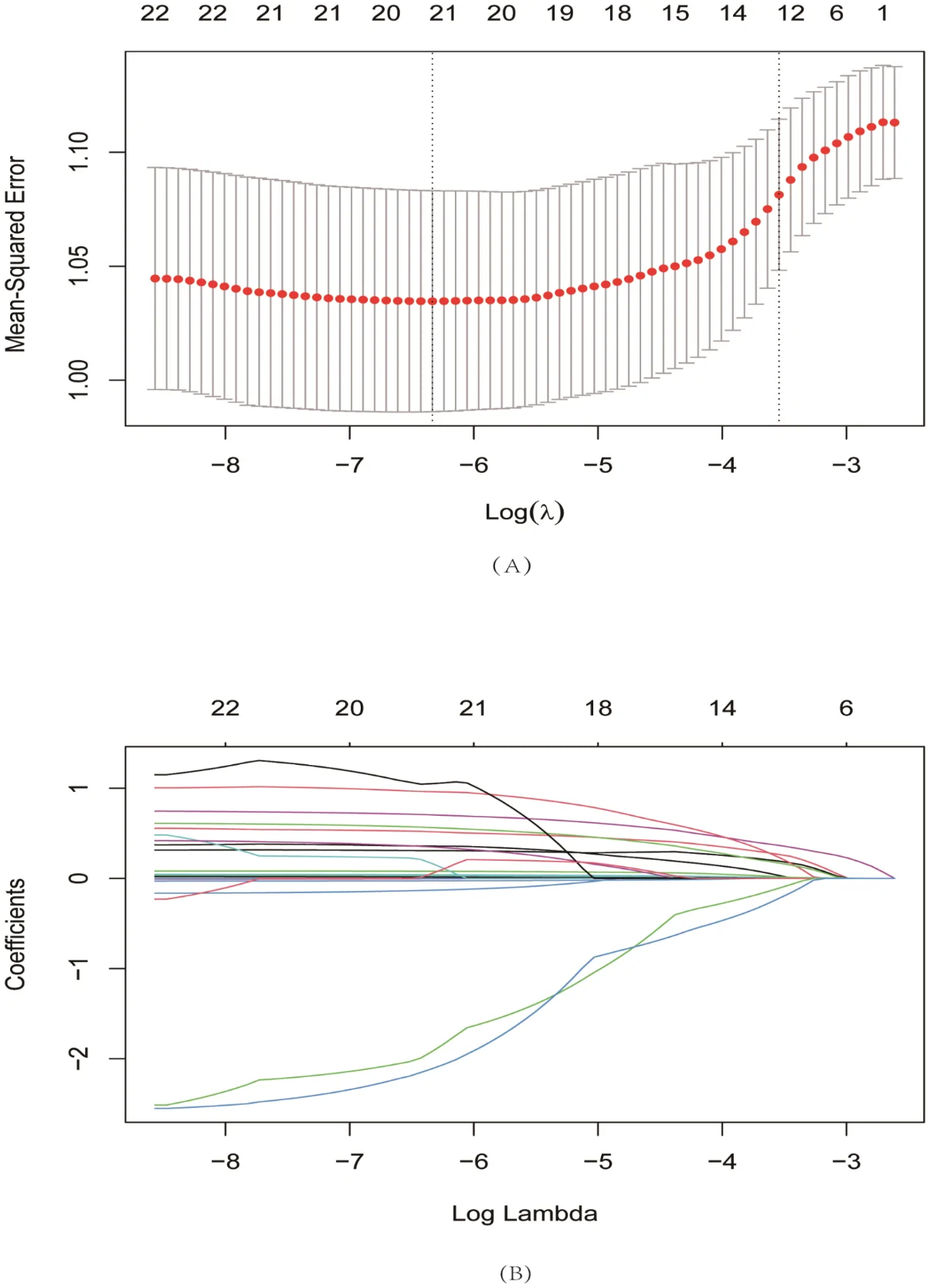
The GS predictive variables were initially screened using the Lasso regression method. **(A)** The optimal parameters were selected through Lasso regression. **(B)** The coefficients corresponding to each variable in the LASSO regression model.

#### 3.3.2 Multivariate logistic regression analysis

Following LASSO screening, 21 candidate predictors were entered into the multivariable logistic regression model. The training cohort included 161 GS events, corresponding to a preselection EPV of 7.7 (161/21). The final six-predictor model had an EPV of 26.8 (161/6). Age (OR = 1.039, 95% CI: 1.019–1.061), HTN (OR = 2.128, 95% CI: 1.360–3.361), FBG (OR = 1.086, 95% CI: 1.021–1.154), TG (OR = 1.536, 95% CI: 1.227–1.923), VLDL-C (OR = 0.060, 95% CI: 0.004–0.367), and ApoA1 (OR = 0.074, 95% CI: 0.015–0.343) met the prespecified P < 0.01 criterion (**Table 2**).

**Table 2:** Results of multivariate logistic regression based on Lasso regression variables.

Variable	Regression coefficient	Standard error	OR value	95% CI	Z value	*P*
Smoke	0.362	0.325	1.436	0.754-2.711	1.113	0.266
Alcohol	0.571	0.335	1.771	0.913-3.414	1.703	0.089
Age	0.039	0.010	1.039	1.019-1.061	3.822	<0.001
Gender	-0.167	0.243	0.846	0.525-1.362	-0.69	0.49
BMI	0.046	0.028	1.047	0.993-1.107	1.677	0.094
HTN	0.755	0.23	2.128	1.36-3.361	3.276	0.001
FLD	0.312	0.225	1.366	0.878-2.128	1.382	0.167
PLG	0.989	0.441	2.69	1.111-6.347	2.242	0.025
CHD	0.621	0.262	1.86	1.11-3.101	2.372	0.018
CD	-0.03	0.015	0.971	0.942-0.999	-1.991	0.047
TBIL	0.027	0.011	1.027	1.007-1.051	2.513	0.012
ALT	-0.008	0.01	0.993	0.972-1.01	-0.77	0.441
AST	0.021	0.014	1.022	0.995-1.051	1.55	0.121
GT	-0.003	0.003	0.997	0.991-1.001	-1.135	0.256
FBG	0.082	0.031	1.086	1.021-1.154	2.647	0.008
TC	0.747	0.827	2.112	0.44-24.179	0.904	0.366
TG	0.429	0.114	1.536	1.227-1.923	3.761	<0.001
HDL-C	0.937	1.208	2.552	0.15-25.408	0.776	0.438
LDL-C	-0.489	0.815	0.613	0.054-2.884	-0.6	0.548
VLDL-C	-2.818	0.963	0.06	0.004-0.367	-2.925	0.003
ApoA1	-2.605	0.79	0.074	0.015-0.343	-3.296	<0.001

#### 3.3.3 Nomogram visualization

A prediction model was constructed based on the six predictors and visualized as a nomogram (**Figure 4**). For individual risk estimation, the value of each predictor is located on the nomogram, and the corresponding points are summed to obtain the total points. The predicted probability of GS is then determined by projecting the total points onto the risk scale.

**Figure 4:**
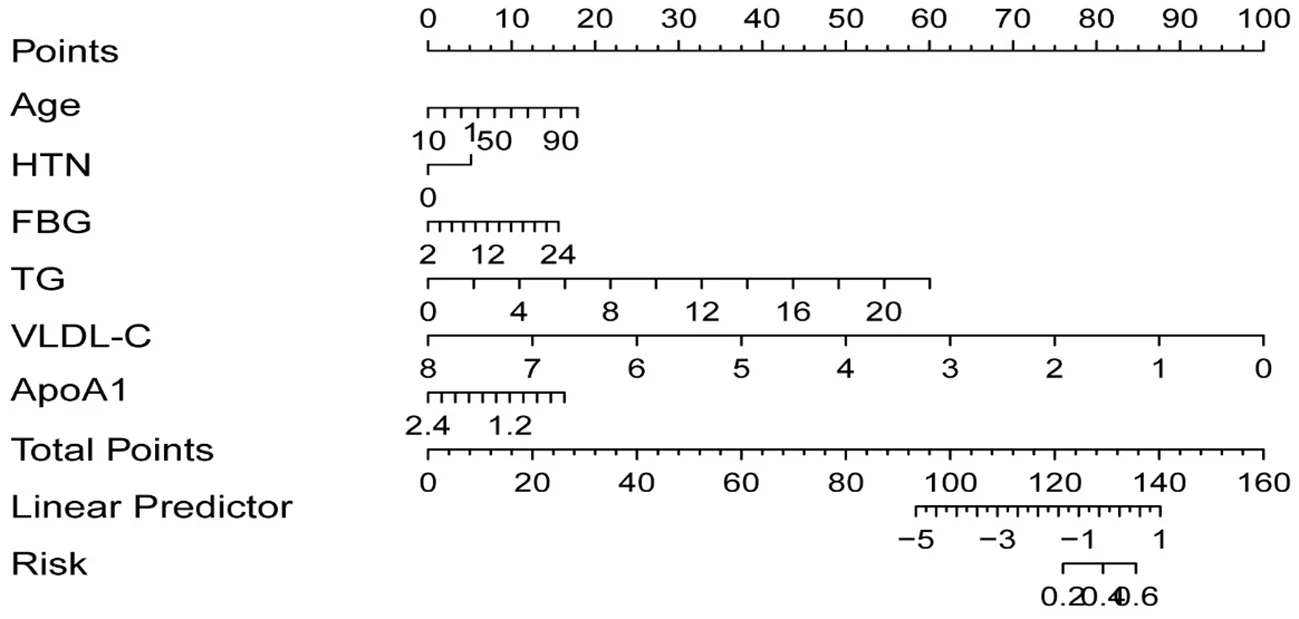
Prediction nomogram for the risk of GS in patients with T2DM.

#### 3.3.4 Discriminative performance

The model showed moderate discrimination. The AUC was 0.713 (95% bootstrap CI 0.669–0.758) in the training cohort and 0.707 (95% bootstrap CI 0.630–0.781) in the internal temporal validation cohort (**Figure 5**).

**Figure 5:**
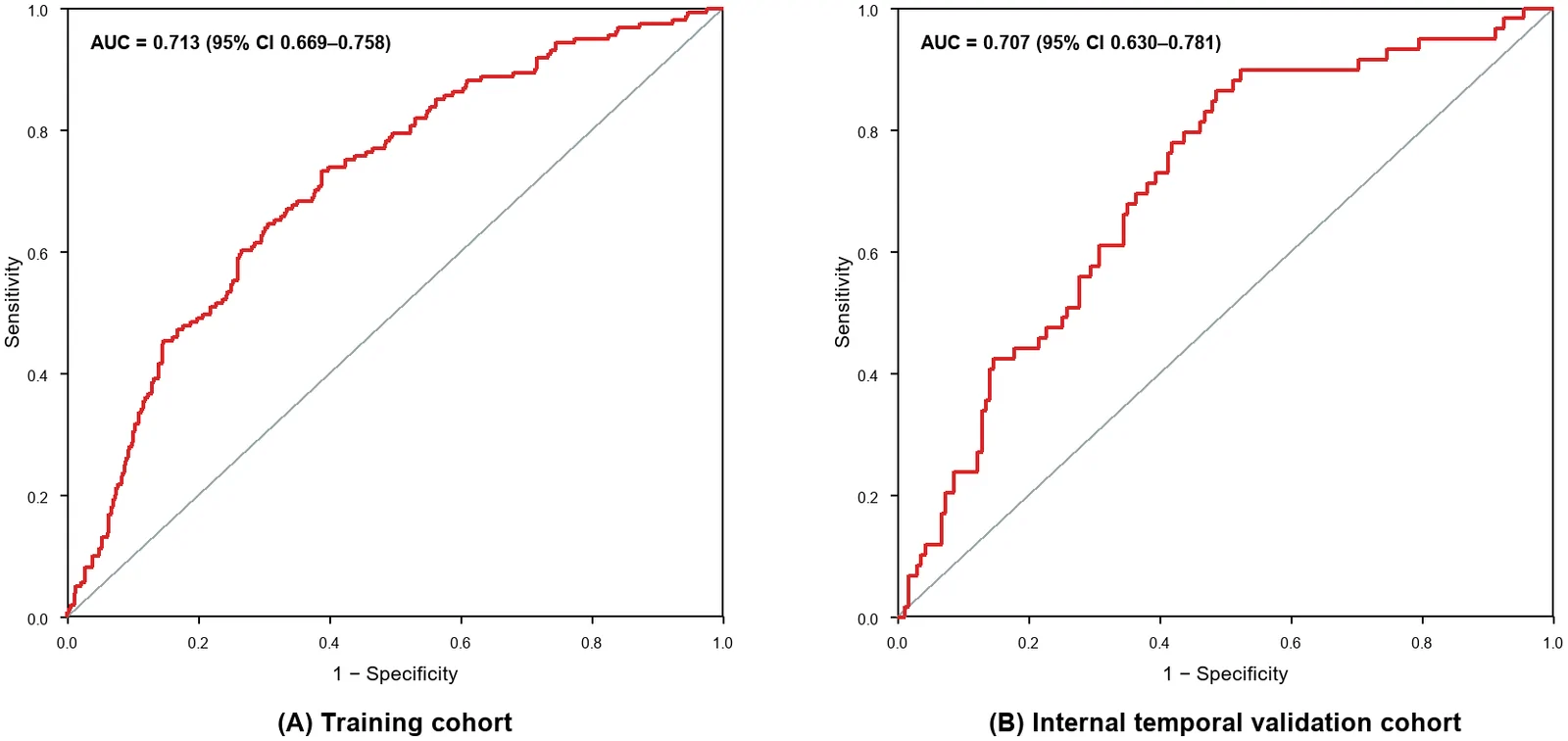
ROC curves of the six-predictor model. **(A)** Training cohort: AUC 0.713 (95% bootstrap CI 0.669–0.758). **(B)** Internal temporal validation cohort: AUC 0.707 (95% bootstrap CI 0.630–0.781).

#### 3.3.5 Model calibration

Calibration was assessed using observed-versus-predicted risk curves, Brier scores, calibration intercepts and slopes, and the Hosmer–Lemeshow test. In the training cohort, the Brier score was 0.166 (95% bootstrap CI 0.150–0.182), the calibration intercept was 0.000 (95% CI −0.318–0.339), and the calibration slope was 1.000 (95% CI 0.745–1.290); the Hosmer–Lemeshow test yielded χ² = 5.813 and P = 0.668. In the internal temporal validation cohort, the Brier score was 0.180 (95% bootstrap CI 0.152–0.210), the calibration intercept was −0.063 (95% CI −0.623–0.573), and the calibration slope was 0.872 (95% CI 0.468–1.455); the Hosmer–Lemeshow test yielded χ² = 9.974 and P = 0.267. The validation point estimates suggest modest overprediction and some compression of calibration, although the bootstrap confidence intervals include the ideal values of 0 and 1 (**Figure 6**).

**Figure 6:**
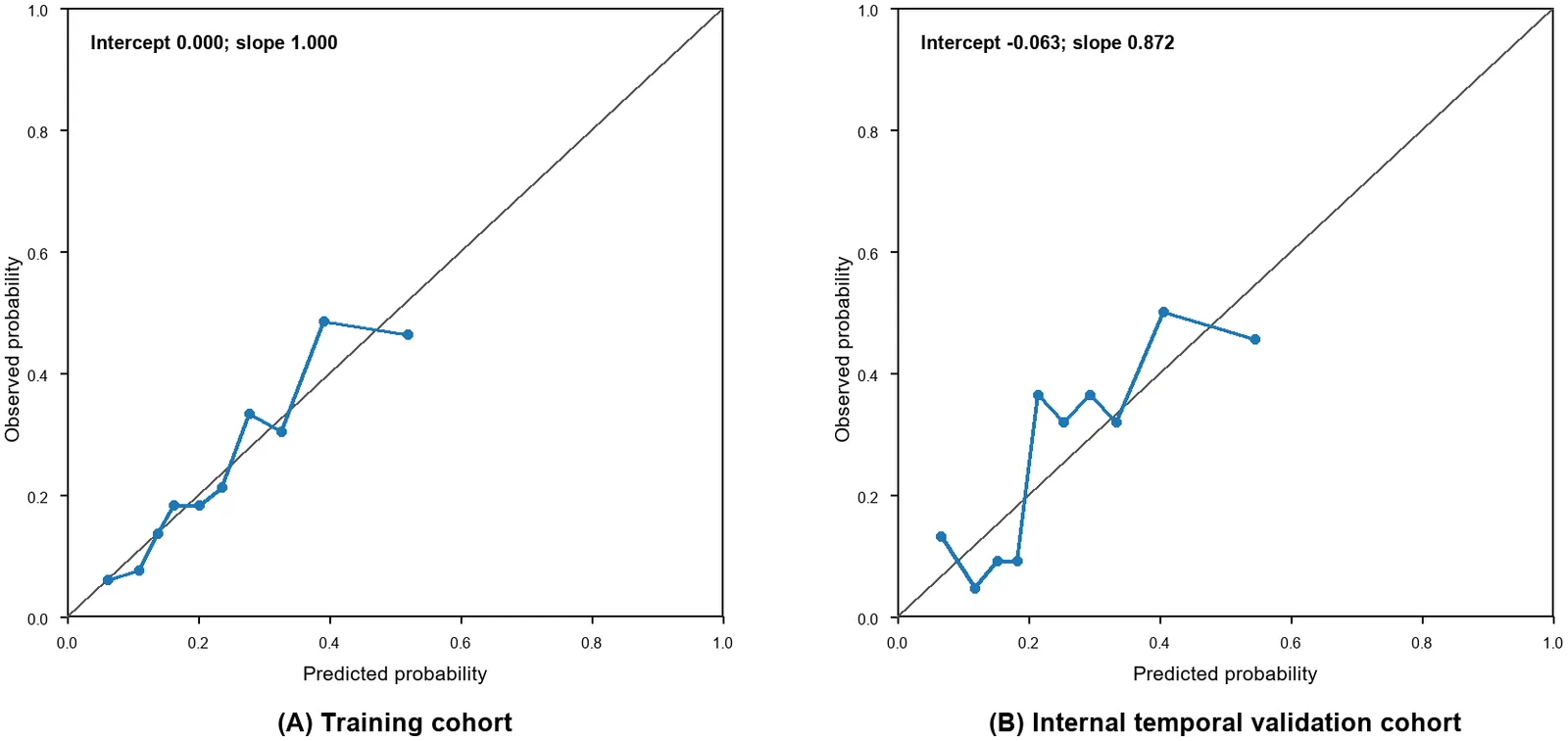
Calibration plots based on observed outcome proportions by predicted-risk decile. **(A)** Training cohort. **(B)** Internal temporal validation cohort.

#### 3.3.6 Decision curve analysis

Potential net benefit was explored using decision curve analysis. The principal contiguous threshold ranges over which the nomogram exceeded both treat-all and treat-none strategies were 8%–44% in the training cohort and 12%–46% in the internal temporal validation cohort. These ranges are sample-dependent decision-analytic estimates rather than validated clinical action thresholds (**Figure 7**).

**Figure 7:**
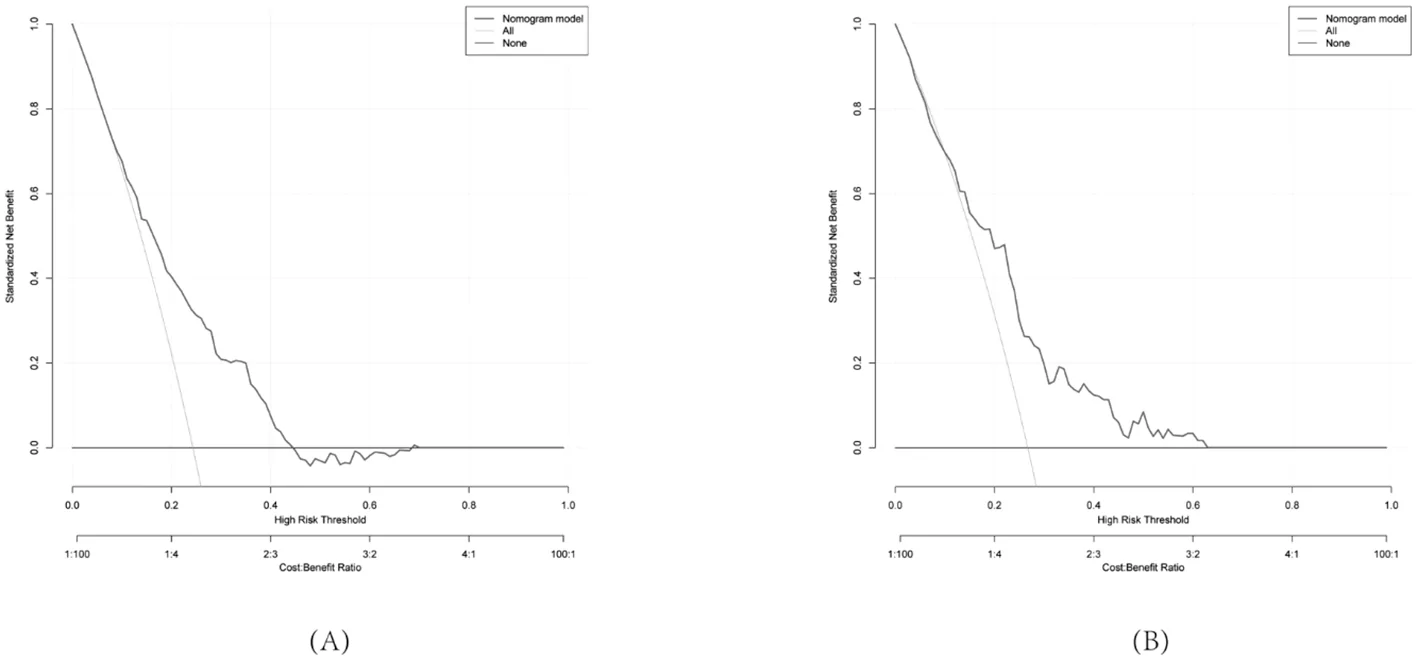
Decision curve analysis of the risk nomogram. **(A)** is the training set, **(B)** is the internal temporal validation cohort. The thick solid line represents the assumption that none of the patients have GS. The thin solid line represents the assumption that all patients have GS. The red solid line represents the risk nomogram.

## 4 Discussion

This study was based on retrospective clinical data and employed LASSO regression combined with multivariable logistic regression to systematically analyze predictors of GS occurrence in patients with T2DM and to construct a risk prediction model. Relative to conventional univariate statistical approaches, this study helped mitigate the influence of multicollinearity on model stability through LASSO-based variable screening and enhanced the robustness of variable selection. The constructed nomogram model not only permits quantitative assessment of candidate predictors but may offer an intuitive adjunctive risk-assessment tool after external validation for clinical practice. The model was validated using multiple complementary metrics including ROC curves, calibration curves, and DCA. The results showed that the model demonstrated moderate discrimination and potential decision-analytic utility, but routine clinical benefit remains unproven.

In our model, VLDL-C emerged as a significant predictor for GS in T2DM patients. Decreased VLDL-C levels were significantly associated with an elevated risk of GS. This finding provides novel evidence supporting the link between diabetic dyslipidemia and GS formation. Previous studies have established dyslipidemia as a major risk factor for GS formation, and patients with T2DM often present with multiple manifestations of lipid metabolism disorders, such as high TG levels, decreased HDL-C, and abnormal VLDL-C (18, 19). Mechanistically, VLDL-C, as a key lipoprotein synthesized and secreted by the liver, mainly participates in the transport of endogenous triglycerides and cholesterol esters to peripheral tissues (20). When its level declines, hepatic cholesterol transport capacity is correspondingly reduced, leading to increased cholesterol flux into bile, thereby elevating biliary cholesterol content and inducing a supersaturated state —a key prerequisite for GS formation (21). In addition to alterations in bile composition, abnormal gallbladder motility also plays an important role in gallstone formation. Diabetes-induced autonomic neuropathy can impair gallbladder contractility and delay emptying, resulting in prolonged bile retention within the gallbladder, thereby creating a favorable environment for the nucleation and aggregation of cholesterol crystals (22). Moreover, insulin resistance, as a core pathophysiological feature of T2DM,promotes hepatic cholesterol synthesis and increases the biliary cholesterol load (23). Additionally, it may influence VLDL-C levels by modulating the activity of HMG-CoA reductase, further elevating GS risk (24, 25). These mechanisms are consistent with our findings.

In the predictive model constructed in this study, TG was also identified as an important indicator affecting GS occurrence. Numerous previous studies have shown that T2DM is closely related to abnormal TG metabolism, and a high TG state can participate in GS formation through various pathways, including influencing hepatic cholesterol metabolism, altering bile composition, and interfering with gallbladder dynamics (26). From a mechanistic perspective, insulin resistance and subsequent hyperinsulinemia constitute the core pathological basis of T2DM (27, 28). In this context, hepatic insulin resistance not only affects glucose metabolism, but also causes lipid metabolism disorders, manifested as increased TG synthesis in the liver and enhanced VLDL secretion, which in turn leads to elevated serum TG levels (29). The elevated TG level can regulate the SREBP-1c and FOXO1 signaling pathways to promote cholesterol synthesis and upregulate the expression of transport proteins ABCG5/8, thereby increasing the excretion of cholesterol into bile. At the same time, insulin resistance can inhibit the activity of enzymes related to bile acid synthesis (such as CYP7B1), reducing bile acid production, and ultimately leading to an imbalance in the ratio of bile acids and cholesterol, creating a supersaturated state conducive to the formation of GS (23). In addition, dyslipidemia can further aggravate the disorder of bile composition through nuclear receptor-related pathways. Studies have shown that an increase in triglycerides can affect the activity of nuclear receptors such as FXR and LXR, thereby inhibiting the synthesis and secretion of bile acids and increasing the relative content of cholesterol in bile (29). At the same time, a high triglyceride state is often accompanied by increased activity of cholesteryl ester transfer protein and decreased HDL-C levels, resulting in a decline in cholesterol excretion capacity. This further promotes the accumulation of cholesterol in the liver and its excretion into bile, thereby exacerbating the bile over-saturation state (21). Apart from changes in bile components, the increase in triglycerides (TG) may also promote the formation of stones by influencing the local microenvironment of the gallbladder. Studies have shown that in a high TG state, the levels of free fatty acids and TG in bile increase, which can stimulate the secretion of mucin by the gallbladder epithelium. Mucin can act as a nucleation substrate for cholesterol crystals, accelerating the formation and aggregation of crystals, and thereby promoting the occurrence and development of stones (30–32).

ApoA1 is a major structural and functional protein of HDL that participates in cholesterol transport and biliary lipid homeostasis. Its potential relevance to GS in T2DM is therefore biologically plausible, but the present observational data do not establish a causal role (31, 33). Previous studies have linked serum lipid profiles and biliary lipid composition with cholesterol crystallization, while experimental work has described cholesterol transport by gallbladder epithelial cells (32). These findings suggest that ApoA1-related pathways may influence cholesterol crystallization and local biliary cholesterol homeostasis, although the direct effect of ApoA1 on GS formation requires more specific experimental confirmation (32, 35, 36, 38). Accordingly, ApoA1 should be considered a component of a broader cholesterol-transport network rather than as an independently established anti-crystallization factor in the bile environment (33, 36, 38). In the gallbladder epithelium, ApoA1 also plays a significant role. Gallbladder epithelial cells express ATP-binding cassette transporter A1 (ABCA1), which can export cholesterol and phospholipids from the cells to ApoA1, forming HDL and maintaining local cholesterol homeostasis (36, 37). The research conducted by Lee et al. further confirmed that there is an ABCA1-dependent cholesterol excretion pathway in the gallbladder epithelium, and ApoA1 is indispensable in this process (38). Therefore, ApoA1 may participate in reverse cholesterol transport and local gallbladder cholesterol homeostasis. In T2DM, metabolic abnormalities may alter the concentration or function of ApoA1; however, the association between circulating ApoA1 and gallstone presence has not been established consistently in the present evidence base (33, 34). Even if the level of ApoA1 does not significantly decrease, its function may still be impaired by metabolic abnormalities associated with diabetes. The study found that advanced glycation end products (AGEs) in a hyperglycemic environment can undergo non-enzymatic modification of ApoA1, significantly weakening its ability to promote cholesterol efflux mediated by ABCA1, and reducing its anti-inflammatory and antioxidant properties (40). Further experiments have shown that the AGE-modified ApoA1 exhibits a loss of function both structurally and functionally. Not only does it result in a decrease in cholesterol excretion, but it also promotes local inflammatory responses, creating favorable conditions for the formation of cholesterol crystals (41). The dual damage of quantity and function eventually leads to an imbalance in the ratio of cholesterol/bile acids/lecithin in bile, increasing the cholesterol saturation level in bile and accelerating the nucleation and growth of cholesterol crystals.

Age is an important factor influencing the occurrence of GS. Studies have shown that as age increases, the incidence of GS significantly rises. The mechanism mainly involves changes in bile components, decreased gallbladder dynamics, and alterations in overall metabolic status. With increasing age, the secretion of cholesterol in the liver gradually increases, while the synthesis and secretion of bile acids decrease relatively, allowing cholesterol in the bile to easily reach an oversaturated state, thereby forming cholesterol monohydrate crystals, which is an important prerequisite for the formation of GS (42). Meanwhile, the smooth muscle contraction function of the gallbladder in the elderly gradually weakens, and the gallbladder’s emptying capacity decreases, causing the bile to remain in the gallbladder for a longer time, creating favorable conditions for the deposition of cholesterol crystals and the formation of GS (43). Furthermore, as one ages, the body often undergoes metabolic changes such as obesity, insulin resistance, and abnormal lipid metabolism. These factors further affect the composition of bile and the dynamics of the gallbladder, promoting the formation and aggregation of cholesterol crystals, thereby increasing the risk of gallstone formation (7). Age plays a significant role in the occurrence and development of GS through the combined effects of these multiple mechanisms.

An elevated FBG is an important risk factor for the formation of GS. Its mechanism of action involves insulin resistance, changes in bile composition, and abnormal gallbladder dynamics. The hyperglycemic state is often accompanied by insulin resistance, which promotes the synthesis and secretion of cholesterol in the liver, leading to an increase in cholesterol content in bile and causing bile to be in an oversaturated state. This provides favorable conditions for the formation of cholesterol crystals, which is the key basis for the formation of GS (9). Long-term high blood sugar can also damage the autonomic nerve function, affect the contraction ability of the gallbladder smooth muscle, delay the gallbladder emptying, increase the retention time of bile in the gallbladder, thereby promoting the deposition of cholesterol crystals and gradually forming GS (42). Meanwhile, high blood sugar further alters the composition of bile by intensifying inflammatory responses and disrupting lipid metabolism, thereby increasing the risk of gallstone formation (44).

The clinical relevance of this nomogram may extend beyond the estimation of gallstone risk. Because the model integrates age, hypertension, fasting blood glucose, and triglycerides, a high score may identify patients with type 2 diabetes who have a substantial systemic metabolic burden rather than merely an isolated hepatobiliary predisposition. Insulin resistance, dyslipidemia, chronic low-grade inflammation, and vascular dysfunction provide a plausible shared metabolic-vascular framework linking gallstone formation with cardiovascular injury. This pattern is consistent with the shared pathophysiology of metabolic syndrome and type 2 diabetes, in which metabolic disturbances may jointly contribute to hepatobiliary and cardiovascular vulnerability (24, 29, 39, 45). Evidence from populations with obesity-related metabolic liver disease likewise supports an association between metabolic-inflammatory burden and early vascular alterations (45). From this perspective, the detection of gallstones or a high nomogram-predicted gallstone risk may serve as an early clinical warning signal of broader cardiometabolic vulnerability and potential cardiovascular risk. Recognition of this connection could shift clinical attention from isolated prevention of hepatobiliary complications toward integrated prevention and longitudinal monitoring, with intensified management of glycemic control, lipid abnormalities, blood pressure, lifestyle-related factors, and established cardiovascular risk factors. Nevertheless, the nomogram was developed to estimate gallstone risk and should not be interpreted as a direct cardiovascular risk-prediction tool.

Several previous studies have developed prediction tools for gallstone-related outcomes, but their target populations, endpoints, and predictors differ from those of the present study. Shabanzadeh et al. developed a risk-stratification rule among 664 community participants with incidentally detected gallstones, focusing on subsequent clinical events in individuals who already had stones; it therefore addresses progression rather than gallstone occurrence in patients with T2DM^( (46)). Morris-Stiff et al. developed a 10-year model for progression from asymptomatic to symptomatic gallstones in 22,257 general medical patients, with a C-statistic of 0.70 and predictors including female sex, younger age, stone number and size, gallbladder polyps, and chronic hemolytic anemia^( (47)). Esen et al. reported 85.42% accuracy for a machine-learning model based on bioimpedance and laboratory data in 319 participants; however, accuracy at a selected threshold is not directly comparable with AUC-based discrimination, and the case-control design and different outcome definition further limit comparison^( (48)). Our nomogram was developed for gallstone occurrence specifically among patients with T2DM and uses routinely available demographic, comorbidity, and metabolic variables; its AUC of approximately 0.71 indicates moderate discrimination. We therefore did not perform a formal head-to-head or incremental-value analysis, because the published models were developed for different populations and outcomes and their predictor definitions and coefficients were not directly transportable to our cohort. The present data do not establish superiority or incremental clinical value. Such claims require evaluation in a common representative cohort, with prespecified comparator models and assessment of discrimination, calibration, and clinical utility. Future multicenter prospective studies should perform this external validation and assess whether model-based risk stratification improves prevention and longitudinal management. Incorporating lifestyle and treatment-related factors may further improve performance and generalizability.

Potential clinical application. The nomogram is intended for adults with established T2DM who undergo routine clinical assessment and whose gallstone status has not yet been established; it is not validated for non-diabetic adults, children, pregnancy, or post-bariatric populations. In a suitable patient, a clinician could enter age, hypertension status, fasting blood glucose, triglycerides, VLDL-C, and ApoA1 into the nomogram to estimate gallstone risk and to identify patients who may merit more intensive metabolic counseling, review of modifiable risk factors, or closer consideration of hepatobiliary assessment. The score should be used as an adjunct to history, examination, and ultrasonography, not as a diagnostic replacement or an automatic indication for treatment. The decision threshold should be chosen locally according to the consequences of additional assessment and available resources; the threshold ranges observed in the DCA are not universal clinical cutoffs. Low predicted risk should not be used to withhold clinically indicated evaluation. Prospective implementation studies are needed to determine which T2DM subgroups benefit, whether risk-based management improves care, and whether the nomogram provides net benefit beyond usual clinical assessment.

This study has several limitations. First, the retrospective single-center design may have introduced selection bias related to referral patterns, clinical indications for admission, and local diagnostic practice. The study population may therefore differ from adults with T2DM managed in primary care or in other regions, limiting transportability. Second, the retrospective nature of the data means that predictor measurement and gallstone ascertainment were not controlled prospectively; ultrasound-based outcome assessment may be subject to measurement variability, and the observational design does not establish causal relationships. Third, several established risk factors were not consistently recorded, including HbA1c, diabetes duration, antidiabetic medications (particularly GLP-1 receptor agonists and insulin), lipid-lowering therapy, rapid weight loss, dietary habits, physical activity, family history, female reproductive factors, and previous bariatric surgery. Their omission may have produced residual confounding and contributed to the model’s moderate predictive performance. Fourth, the training cohort contained 161 gallstone events and 21 LASSO-retained candidate predictors, corresponding to an approximate preselection EPV of 7.7, below the traditional 10-event heuristic. Although the final six-predictor model had an EPV of 26.8 and internal bootstrap validation was performed, the preselection-stage EPV and the data-driven selection process mean that overfitting cannot be excluded. The stability of the selected predictors also requires confirmation because repeated resampling-based selection-frequency analysis was not performed. Fifth, the later cohort came from the same institution and represents internal temporal validation rather than independent external validation. Thus, geographic, healthcare-system, and population heterogeneity were not tested, and the model’s generalizability remains uncertain. Sixth, the DCA results estimate potential decision-analytic net benefit in these cohorts but do not establish a universal clinical threshold, improved patient outcomes, or benefit from implementation. Finally, no prospective implementation study assessed whether use of the nomogram changes clinician behavior or improves patient care. Larger multicenter prospective studies should prespecify the candidate predictors, collect the currently unavailable risk factors, assess measurement and calibration rigorously, externally validate the model, examine predictor stability, and evaluate clinical utility before routine implementation.

## 5 Conclusion

1. Older age, HTN, elevated FBG, increased TG, decreased VLDL-C, and decreased ApoA1 were independent predictors of GS occurrence in patients with T2DM.
2. The nomogram showed moderate discrimination and may support risk stratification among patients with T2DM; independent external validation and prospective impact assessment are required before routine implementation.
3. In clinical practice, the detection of GS or a high nomogram-predicted GS risk may serve as an early warning signal of substantial systemic metabolic burden, broader cardiometabolic vulnerability, and potential cardiovascular risk. This interpretation supports a shift from isolated hepatobiliary prevention toward integrated metabolic and cardiovascular risk management and longitudinal monitoring, including glycemic control, lipid abnormalities, blood pressure, lifestyle-related factors, and hepatobiliary risk.
4. LASSO regression helped mitigate the influence of multicollinearity during variable screening, and the constructed nomogram provides a quantitative approach to estimate GS risk that may serve as an adjunct to early clinical risk stratification after further validation.

## Data Availability

The original contributions presented in the study are included in the article/**Supplementary Material**. Further inquiries can be directed to the corresponding author.

## Supplementary material

Supplementary Table 1 TRIPOD checklist for the prediction model study.
